# Partial dependence of ultrasonically estimated fetal weight on biometric parameters

**DOI:** 10.1098/rsos.250172

**Published:** 2025-06-18

**Authors:** Vasiliki Bitsouni, Nikolaos Gialelis, Vasilis Tsilidis

**Affiliations:** ^1^ Department of Mathematics, University of Patras, Patras, Greece; ^2^ Department of Mathematics, National and Kapodistrian University of Athens, Athens, Attica, Greece; ^3^ School of Medicine, National and Kapodistrian University of Athens, Athens, Attica, Greece

**Keywords:** sobol’ method, bootstrapping, ultrasound formula, estimation of fetal weight, head circumference, abdominal circumference

## Abstract

Accurate assessment of estimated fetal weight (EFW) is crucial in obstetrics, yet the exact contribution of biometric parameters in sonographic formulas remains unclear. Twenty-six datasets from published studies spanning diverse populations and gestational ages were analysed, incorporating measurements of biparietal diameter (BPD), abdominal circumference (AC), head circumference (HC) and femur length (FL). Sobol’ global sensitivity analysis—a variance‑based approach—quantified each parameter’s influence on EFW across 29 established formulas, and bootstrapping estimated the median of the sensitivity indices with 95% confidence intervals. Results showed that AC was generally the dominant predictor, especially in later pregnancy, while BPD, HC and FL exhibited variable importance depending on formula and gestational age. Two-thirds of the formulas demonstrated parameter crossover effects, and nearly half had at least one parameter with minimal contribution. These findings indicate that parameter significance differs by both formula and gestational age, suggesting that clinicians should select EFW formulas based on gestational age, measurement reliability and fetal characteristics. Estimates made with fewer than the intended parameters can be viable in emergencies. The proposed methodology can guide the refinement of existing formulas and the development of improved fetal weight estimation models.

## 1. Introduction

**Figure 1.**
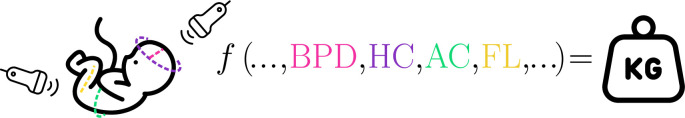
The biometric characteristics of a fetus, such as the circumference of its head and abdomen, the length of its femur, or its biparietal diameter are measured using ultrasound. The measurements are then entered into various mathematical formulas to estimate the weight of the fetus.

Estimating the weight of a fetus is a critical aspect of prenatal care, primarily conducted through ultrasound imaging [1–4]. This method allows healthcare providers to measure various fetal parameters, such as the biparietal diameter (BPD), abdominal circumference (AC), head circumference (HC) and femur length (FL). These measurements are then used as parameters in established mathematical formulas to calculate the estimated fetal weight (EFW) [5] (see figure 1).

The parameters of a mathematical function do not necessarily contribute equally to its output. In many mathematical models, particularly nonlinear ones, certain parameters can have a more significant impact on the result than others. Mathematical methods, such as sensitivity analysis, can reveal the varying degrees of influence each parameter has, illustrating that the overall result is often a product of both individual and interactive effects among the parameters involved [6,7]. This complexity underscores the importance of analysing parameter contributions rather than assuming uniform influence across all inputs.

As pregnancy progresses, the accuracy of fetal head measurement becomes increasingly challenging. This is due to various factors, including the position of the fetus, the volume of amniotic fluid and the presence of an anterior placenta. Furthermore, the fetal head may engage with the pelvis, potentially impeding the acquisition of clear ultrasound images [8–10]. Therefore, in the latter stages of pregnancy, formulas that are influenced less by head parameters (BPD and HC) could lead to more accurate predictions.

Shaheen *et al*. [11] conducted a comparative analysis of two distinct fetal weight estimation formulas, one incorporating HC and one that does not. The study concluded that the discrepancy in accuracy between these formulas was negligible. Consequently, the researchers deduced that, in the context of this particular comparison, HC does not constitute a substantial parameter.

Is it possible to extend the conclusions drawn by Shaheen *et al.* [11], i.e. to systematically analyse the contributions of parameters across different formulas? Establishing a way to quantify the contribution of each parameter in fetal weight estimation formulas would enable comparisons and resolve the aforementioned inquiry. Sensitivity analysis emerges as a mathematical approach that can effectively tackle this challenge [6,7]. Techniques such as the Sobol’ method [12] are used in sensitivity analysis to measure the extent to which a parameter influences the output of a given problem, quantified by a metric known as the Sobol’ index.

There exist many studies comparing the accuracy of different formulas for the calculation of EFW [3,5,13–20]. They often involve retrospective and prospective analysis, comparing predicted weights to actual birth weights to assess accuracy, bias and reproducibility. Such articles underscore the ongoing quest for more reliable fetal weight estimation techniques that can enhance obstetric care by improving predictions related to delivery management and neonatal outcomes. However, no research has been conducted that systematically assesses the contribution of the involved parameters of each formula to the EFW.

The scope of the present study is to evaluate the effect of the biometric parameters of various formulas on the EFW. The utilized approach relies on an unbiased global sensitivity analysis scheme, known as the Sobol’ method.

## 2. Methods

**Figure 2.**
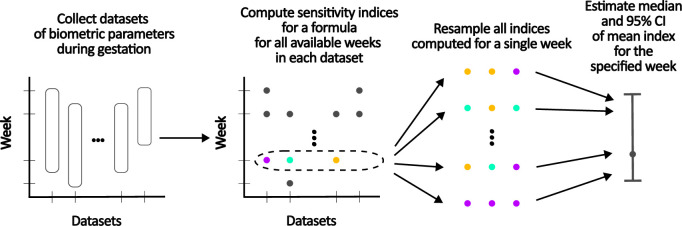
The current study deploys a multi-step process involving the utilization of numerous datasets sourced from existing literature. The Sobol' method is then applied to each of these datasets, for all the studied formulas. Following this, bootstrapping is conducted using the computed Sobol' indices from each individual week as the sample points (the figure uses colour to visualize the different sample points). Finally, the median, along with a 95% confidence interval, are estimated from the mean Sobol' index of the resamples.

An overview of the implemented methods can be found in figure 2 and a detailed description is presented below.

### 2.1. Formulas

The formulas under investigation, which take into account more than one independent parameter, are presented in table 1. These formulas take a combination of BPD, AC, HC and FL as their parameter arguments.

**Table 1.** List of the formulas under investigation.

source	formula
Combs [21]	$0.23718{\left(\text{AC}\right)}^{2}\left(\text{FL}\right)+0.03312{\left(\text{HC}\right)}^{3}$
Ferrero [22]	$10^{0.77125+0.13244\left(\text{AC}\right)-0.12996\left(\text{FL}\right)-1.73588{\left(\text{AC}\right)}^{2}/1000+3.09212\left(\text{AC}\right)\left(\text{FL}\right)/1000+2.18984\left(\text{FL}\right)/\left(\text{AC}\right)}$
Hadlock I [23]	$10^{1.304+0.05281\left(\text{AC}\right)+0.1938\left(\text{FL}\right)-0.004\left(\text{AC}\right)\left(\text{FL}\right)}$
Hadlock II [23]	$10^{1.335-0.0034\left(\text{AC}\right)\left(\text{FL}\right)+0.0316\left(\text{BPD}\right)+0.0457\left(\text{AC}\right)+0.1623\left(\text{FL}\right)}$
Hadlock III [23]	$10^{1.326-0.00326\left(\text{AC}\right)\left(\text{FL}\right)+0.0107\left(\text{HC}\right)+0.0438\left(\text{AC}\right)+0.158\left(\text{FL}\right)}$
Hadlock IV [23]	$10^{1.3596+0.00061\left(\text{BPD}\right)\left(\text{AC}\right)+0.424\left(\text{AC}\right)+0.174\left(\text{FL}\right)+0.0064\left(\text{HC}\right)-0.00386\left(\text{AC}\right)\left(\text{FL}\right)}$
Halaska [24]	$10^{0.64041\left(\text{BPD}\right)-0.03257{\left(\text{BPD}\right)}^{2}+0.00154\left(\text{AC}\right)\left(\text{FL}\right)}$
Hsieh [25]	$10^{2.1315+0.0056541\left(\text{BPD}\right)\left(\text{AC}\right)-0.00015515\left(\text{BPD}\right){\left(\text{AC}\right)}^{2}+0.000019782{\left(\text{AC}\right)}^{3}+0.052594\left(\text{BPD}\right)}$
INTERGROWTH-21 [26]	$e^{5.084820-54.06633{\left(\left(\text{AC}\right)/100\right)}^{3}-95.80076{\left(\left(\text{AC}\right)/100\right)}^{3}\ln\left(\left(\text{AC}\right)/100\right)+3.136370\left(\text{HC}\right)/100}$
Jordaan I [27]	$10^{-1.1683+0.0377\left(\text{AC}\right)+0.095\left(\text{BPD}\right)-0.0015\left(\text{BPD}\right)\left(\text{AC}\right)}$
Jordaan II [27]	$10^{0.9119+0.0488\left(\text{HC}\right)+0.0824\left(\text{AC}\right)+0.001599\left(\text{AC}\right)\left(\text{HC}\right)}$
Merz [28]	$3200.40479+157.07186\left(\text{AC}\right)+15.90391{\left(\text{BPD}\right)}^{2}$
Ott [29]	$10^{-2.0661+0.04355\left(\text{HC}\right)+0.05394\left(\text{AC}\right)-0.0008582\left(\text{AC}\right)\left(\text{HC}\right)+1.2594\left(\text{AC}\right)\left(\text{FL}\right)\left(\text{AC}\right)}$
Roberts [30]	$10^{1.6758+0.01707\left(\text{AC}\right)+0.042478\left(\text{BPD}\right)+0.05216\left(\text{FL}\right)+0.01604\left(\text{HC}\right)}$
Schild [31]	$5381.193+150.324\left(\text{HC}\right)+2.069{\left(\text{FL}\right)}^{3}+0.0232{\left(\text{AC}\right)}^{3}-6235.478\;\log(\text{HC})$
Shepard I [32]	$10^{-1.599+0.144\left(\text{BPD}\right)+0.032\left(\text{AC}\right)-0.000111\left(\text{AC}\right){\left(\text{BPD}\right)}^{2}}$
Shepard II [32]	$10^{-1.7492+0.166\left(\text{BPD}\right)+0.046\left(\text{AC}\right)-0.002646\left(\text{BPD}\right)\left(\text{AC}\right)}$
Thurnau [33]	9.337(BPD)(AC)−229
Vintzileos I [34]	$10^{1.879+0.084\left(\text{BPD}\right)+0.026\left(\text{AC}\right)}$
Vintzileos II [34]	$10^{2.082+0.004\left(\text{HC}\right)\left(\text{FL}\right)+0.0018\left(\text{AC}\right)-0.00000001509{\left(\left(\text{HC}\right)\left(\text{FL}\right)\right)}^{3}}$
Warsof [35]	$10^{-1.599+0.144\left(\text{BPD}\right)+0.032\left(\text{AC}\right)-0.000111{\left(\text{BPD}\right)}^{2}\left(\text{AC}\right)}$
Weiner I [36]	$10^{1.6961+0.02253\left(\text{HC}\right)+0.01645\left(\text{AC}\right)+0.06439\left(\text{FL}\right)}$
Weiner II [36]	$10^{1.6575+0.04035\left(\text{HC}\right)+0.01285\left(\text{AC}\right)}$
Woo [37]	1.4(BPD)(AC)(HC)−200
Woo I [38]	$10^{1.54+0.15\left(\text{BPD}\right)+0.00111{\left(\text{AC}\right)}^{2}-0.0000764\left(\text{BPD}\right){\left(\text{AC}\right)}^{2}+0.05\left(\text{FL}\right)-0.000992\left(\text{AC}\right)\left(\text{FL}\right)}$
Woo II [38]	$10^{1.14+0.16\left(\text{BPD}\right)+0.05\left(\text{AC}\right)-2.8\left(\text{BPD}\right)\left(\text{AC}\right)/1000+0.04\left(\text{FL}\right)-4.9\left(\text{AC}\right)\left(\text{FL}\right)/10000}$
Woo III [38]	$10^{1.13+0.18\left(\text{BPD}\right)+0.05\left(\text{AC}\right)-3.35\left(\text{BPD}\right)\left(\text{AC}\right)/1000}$
Woo IV [38]	$10^{1.63+0.16\left(\text{BPD}\right)+0.00111{\left(\text{AC}\right)}^{2}-0.0000859\left(\text{BPD}\right){\left(\text{AC}\right)}^{2}}$
Woo V [38]	$10^{0.59+0.08\left(\text{AC}\right)+0.28\left(\text{FL}\right)-0.00716\left(\text{AC}\right)\left(\text{FL}\right)}$

### 2.2. Sensitivity analysis

The Sobol’ method [12], a global sensitivity analysis technique, is a variance-based approach used to evaluate how changes in parameter inputs influence the outputs of a mathematical model. The method operates by partitioning the variance of the outputs generated by the model into distinct contributions from individual input parameters and their respective interactions [6,7]. Assuming that f is the mathematical formula used for fetal weight estimation and X is the vector of the parameters of f, then by the Hoeffding decomposition, the total variance of the formula can be written as


$$\text{Var}[f(\text{X})]=\sum_{i=1}^{d}V_{i}+\sum_{i<j}^{d}V_{i,j}+\cdots +V_{1,2\ldots ,d},$$

where $V_{i}$ represents the variance due to the main effect of parameter $X_{i}$, $V_{i,j}$ represents the variance due to the interaction between parameters $X_{i}$ and $X_{j}$ and so on.

Sobol’ sensitivity indices are normalized metrics that correspond to the aforementioned representations. The first-order sensitivity indices are defined as


$$S_{i}=\frac{V_{i}}{\text{Var}\left[f(\text{X})\right]}.$$

A high first-order index indicates that the parameter has a significant direct influence on the output.

The second-order sensitivity indices are defined as


$$S_{i,j}=\frac{V_{i,j}}{\text{Var}\left[f(\text{X})\right]}.$$

A high second-order index indicates that the combined effects of the two parameters are not merely additive. Both the first- and second-order indices range from 0 to 1.

Higher order indices are calculated similarly.

The total-order indices are defined as


$$S_{T_{i}}=S_{i}+\sum_{j\neq i}S_{i,j}+\sum_{j<k\neq i}S_{i,j,k}+\ldots \;.$$

It captures the total contribution of parameter $X_{i}$, including its main effect and all interactions with other variables. Unlike the nth order indices, they do not range from 0 to 1.

The Julia package GlobalSensitivity.jl [39] is utilized for the calculation of the Sobol’ indices. The variances are calculated numerically using the Jansen estimator [40].

### 2.3. Data

**Figure 3.**
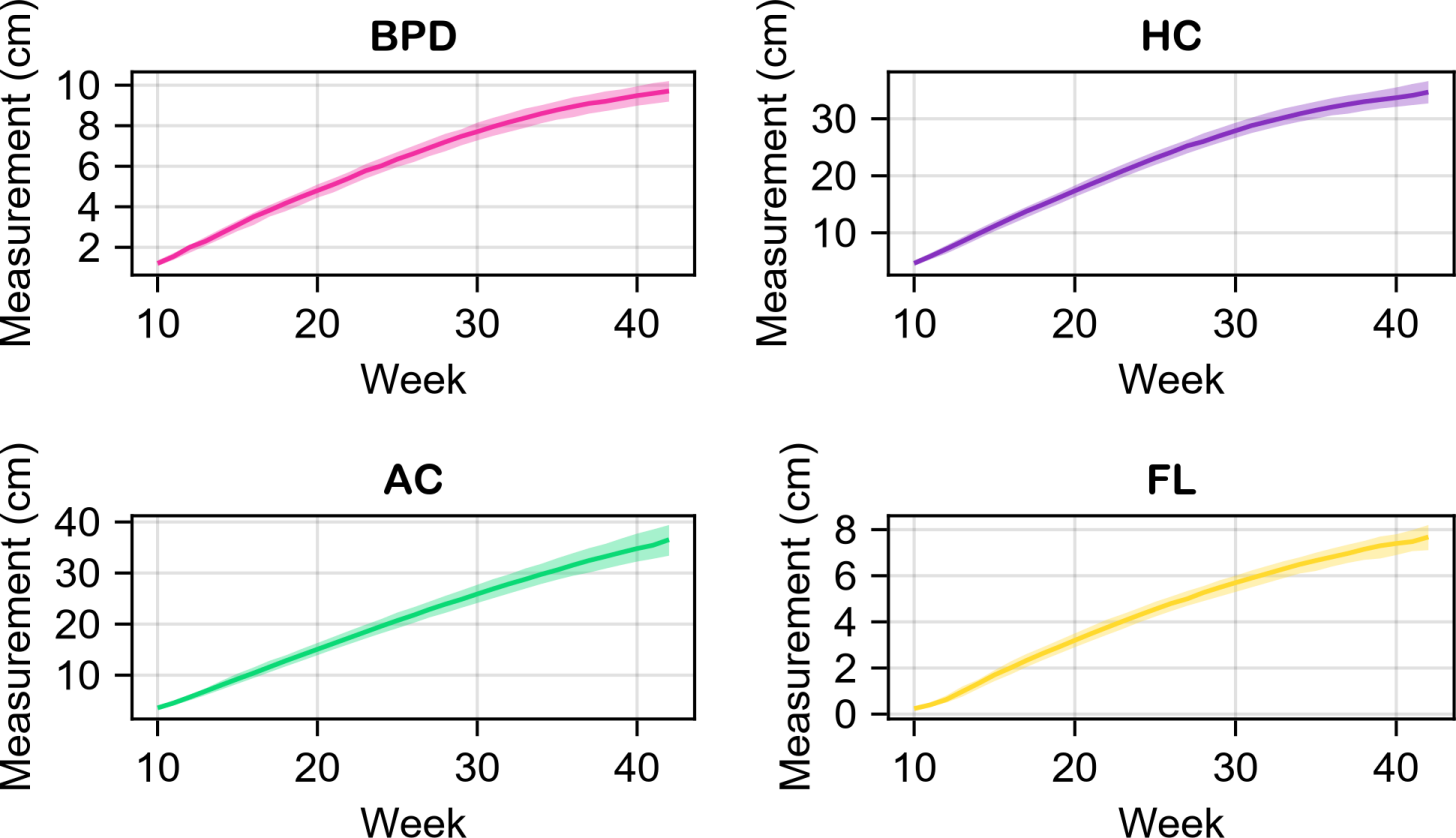
Descriptive statistics pertaining to the bounds of the biometric parameters of interest. The solid lines represent the median corresponding to the 50th percentile, whereas the bands illustrate the medians of the 10th and 90th percentiles, respectively, based on the aggregated data obtained from the sources outlined in table 2.

A rundown of the utilized datasets can be seen in table 2. Descriptive statistics for BPD, HC, AC and FL are presented in figure 3. An in-depth look at the datasets is provided in the electronic supplementary material. In all the studies presented in table 2, a number of measurements, regarding the parameters of interest, were taken from pregnant women of various ethnicities at various gestational ages. Furthermore, the researchers calculated the 10th and 90th percentile charts for all parameters and for all gestational ages investigated in each study. The present study examines the 10th and 90th percentiles of each parameter on a weekly basis, designating these values as the lower and upper bounds for each parameter during each week. These bounds are critical for the parameter sampling that is required for the Sobol’ method.

**Table 2.** Rundown of the utilized datasets. Further details are included in the electronic supplementary material.

source	year	country	number of fetuses	weeks
Araujo Júnior *et al.* [41]	2014	Brazil	31 476	18–38
Brons *et al.* [42]	1990	The Netherlands	63	12–40
Browne *et al.* [43]	1992	USA	8285	10–44
Buck Louis *et al.* [44] (Asian)	2015	USA	460	10–40
Buck Louis *et al.* [44] (Black)	2015	USA	611	10–40
Buck Louis *et al.* [44] (Hispanic)	2015	USA	649	10–40
Buck Louis *et al.* [44] (White)	2015	USA	614	10–40
Chitty *et al.* [45–47]	1994	UK	594–649	12–42
de la Vega *et al.* [48]	2008	Puerto Rico	548	14–38
Dubiel *et al.* [49]	2008	Poland	959	20–42
Dulger *et al.* [50]	2024	Turkey	1132	15–40
Fouad *et al.* [51]	2021	Egypt	540	14–40
Giorlandino *et al.* [52]	2009	Italy	4896	14–41
Johnsen *et al.* [53]	2006	Norway	650	10–42
Jung *et al.* [54]	2007	South Korea	10 455	12–40
Kiserud *et al.* [55]	2017	multinational	1387	14–40
Kwon *et al.* [56]	2014	South Korea	986	15–40
Lai & Yeo [57]	1992	Singapore	6374	14–41
Lessoway *et al.* [58]	1998	Canada	1396	11–42
Lindström *et al.* [59]	2020	Sweden	583	12–42
Munim *et al.* [60]	2012	Pakistan	1228	20–39
Paladini *et al.* [61]	2005	Italy	626	16–40
Papageorghiou *et al.* [62]	2014	multinational	13 108	14–40
Saksiriwuttho *et al.* [63]	2007	Thailand	628	14–41
Stampalija *et al.* [64]	2020	Italy	7347	15–40
Verburg *et al.* [65]	2008	The Netherlands	8313	12–40

### 2.4. Sampling

The computation of variances using the Jansen estimator necessitates the use of two design matrices. In order to construct a design matrix, it is essential to have a method for generating samples of the parameters.

In order to eliminate the bias from the dataset and at the same time investigate the whole parameter space uniformly (i.e. more evenly), it is assumed that, for each week, the parameters follow a continuous uniform distribution, between the bounds defined by the 10th and 90th percentiles charts of that week. To this end, a quasi-random low-discrepancy sequence of numbers is generated in the interval between the 10th and the 90th percentile of each parameter, utilizing the Sobol’ sampling method, which is described in Sobol' [12]. The Julia package QuasiMonteCarlo.jl is used to generate design matrices consisting of Sobol’ sequences, with a sample size of $10^{6}$, for each formula and each week.

### 2.5. Estimation of the mean of indices with bootstrapping

The mean values of the indices of each formula are estimated using non-parametric bootstrapping, which is described in Casella & Berger [66]. This method is chosen due to its no distributional assumptions, robustness to outliers and usefulness in small samples.

To obtain the median, along with a 95% CI for the estimation of the mean of the Sobol’ indices, the methodology outlined below is followed.

(i) The Sobol’ indices are calculated for every combination of formula and dataset for each week in which data are available.
(ii) The calculated Sobol’ indices are grouped together by formula and gestation age (excluding weeks 43 and 44 due to scarcity of available data).
(iii) $10^{6}$ bootstrap samples are created for each aforementioned group.
(iv) The average is calculated for each bootstrap sample, resulting in samples of size $10^{6}$ for the respective mean value of each group.
(v) The median, along with a 95% CI, is estimated for each mean-value sample, using the empirical distribution function.

## 3. Results

Figure 3 presents an estimate of the first-order Sobol’ indices for a curated list of formulas of table 1, based on the datasets of table 2. Each panel consists of an error bar for each parameter and for each gestational week. The median of the mean value of the indices is represented by a dot, while the whiskers represent a 95% CI. In addition, each panel displays the indices for each individual dataset as a scatter plot with a low alpha value. The complete list of obtained results, for all formulas of table 1, is presented in electronic supplementary material, figures S1-S29.

**Figure 4.**
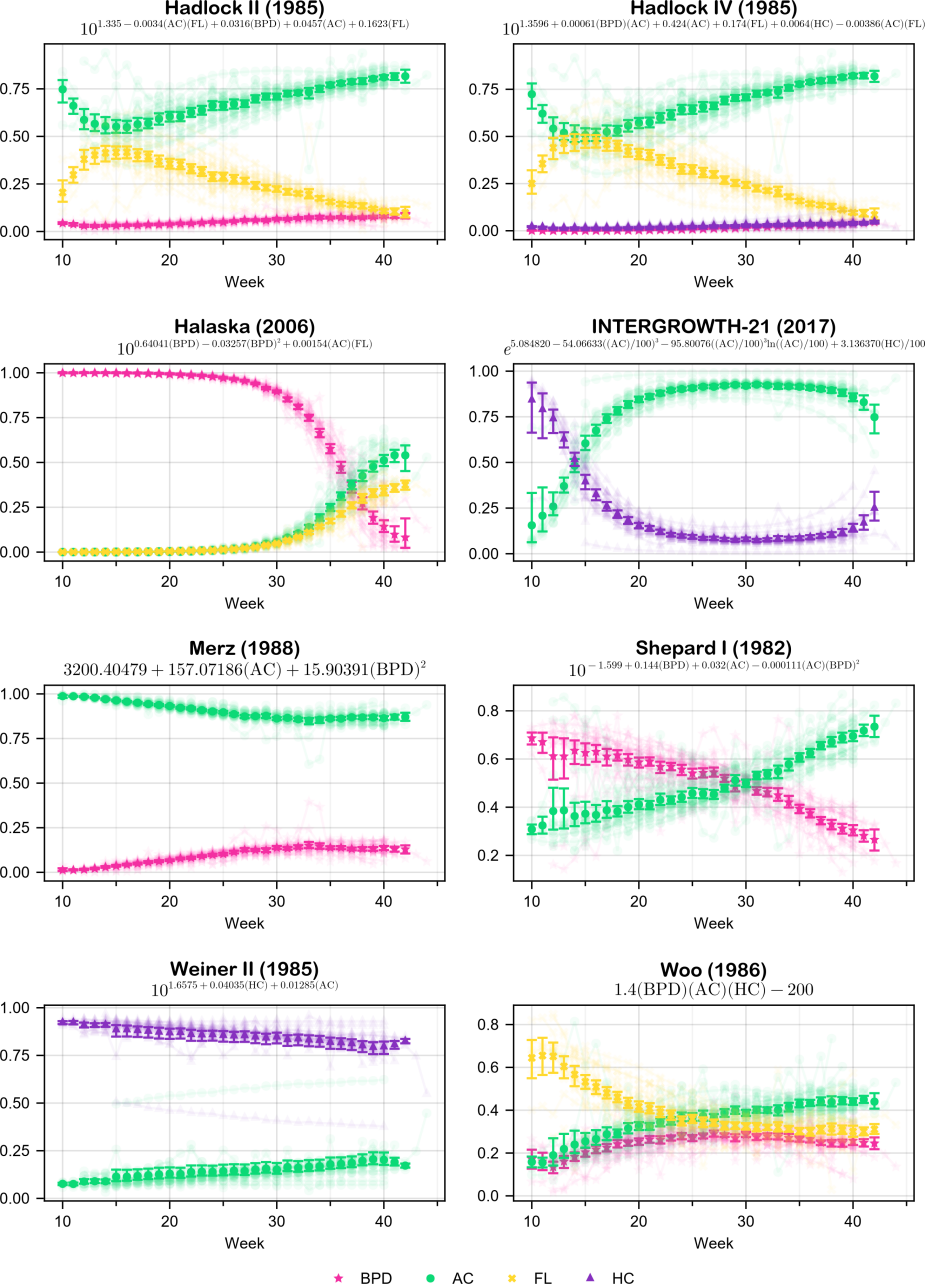
Comparison of the first-order Sobol' index (measure of parameter significance) of different fetal weight estimation models over gestational week 10 through 42. The figure presents an estimate of the median (solid markers) and a 95% CI (error bars) of the mean Sobol' index computed for each formula and gestational age, for various datasets. Each panel corresponds to a different formula, with the equation used for estimation displayed at the top of each plot. The colour legend indicates the biometric parameters used in the models: biparietal diameter (BPD, pink), abdominal circumference (AC, green), femur length (FL, yellow) and head circumference (HC, purple). The indices for each individual dataset are illustrated in each panel as scatter plots, utilizing a low alpha value. The data suggest varying trends across models, with some showing diverging patterns of relative contribution from different parameters.

The higher order Sobol’ indices for all the tested formulas were found to be small (< 0.01). Hence, they are not presented. Given the insignificance of the higher order indices, the total-order indices are approximately equal to the first-order indices. Therefore, they are also omitted.

In terms of a general overview of the results, approximately half of the formulas (45%) have at least one parameter with a first-order Sobol’ index that is less than 0.3.

Moreover, 66% of the formulas exhibit a crossover in parameter importance—some transition from low-to-high significance, while others decline from high to low—over the course of gestation. This indicates that in most formulas, the parameters undergo a reversal in their significance level.

With regard to the monotonicity of the indices, it is observed that, following the 20th week, the vast majority of the studied formulas exhibit strictly monotonic indices. During weeks 10–20, approximately 41% of the formulas exhibit at least one index with a turning point. Notably, all of these formulas contain FL as a parameter. Furthermore, only two of the 16 formulas with FL as a parameter do not display a turning point (12.5%).

With respect to the importance of each parameter, the presence of HC is insignificant (having an index less than 0.3 throughout the whole gestation) in 20% of the formulas that it appears in, BPD in 33%, AC in 4% and FL in 38%. On the contrary, only two of the formulas (7%) have a consistently dominant index, with values greater than 0.7 throughout the gestation. Specifically, HC is dominant in Weiner II (1985) and AC is dominant in Merz (1988).

As far as the uncertainty of the estimates is concerned, the length of the 95% CI is generally narrow, indicating a precise estimate. However, some formulas have a more broad 95% CI during weeks 10–15, such as INTERGROWTH-21 (2017) and Shepard I (1982), as well as during weeks 41 and 42, such as INTERGROWTH-21 (2017) and Halaska (2006).

## 4. Discussion

### 4.1. Summary of key findings

This study demonstrated that nearly half of the formulas under investigation include at least one parameter which admits a very low Sobol’ index. This indicates that a considerable number of the formulas are not parsimonious, as they utilize more parameters than are necessary to make a prediction for the EFW. Similar findings have been observed in previous studies, such as the insignificance of HC in Hadlock III (1985) [11].

AC is considered to be a crucial parameter in fetal weight estimation [67–71]. After all, it is included in all the studied formulas. The results of the present study highlight the importance of AC; however, just like all the tested parameters, its contribution depends on both the choice of the formula and the gestation age.

Furthermore, two-thirds of the formulas exhibit a crossover effect, with at least two of the parameters overtaking each other over time. This noteworthy phenomenon can be attributed to various factors. To begin with, as can be seen from figure 3, during week 10 the aggregate measurements of all the biometric parameters show a standard deviation of around 2cm. This standard deviation continues to increase throughout gestation, reaching about 15.5cm by the 42nd week. The substantial increase in the variation of inter-parameter values may elucidate the crossover effect observed in the indices, given the considerable differences in parameter sizes. Another potential explanation lies in the way the formulas are generated. The regression analysis used for the creation of the formulas does not take into account the temporal dependencies of the parameters. A thorough search of the relevant literature yielded no formulas that are produced utilizing time series regression, and it would be worthwhile to examine if such a model could admit time-stable indices.

Regarding the second-order Sobol’ indices, they measure the degree of interaction between the associated parameters. The term ‘interaction’ is used to describe a situation in which the effect of one variable on an outcome depends on the level of another variable. Consequently, if the second-order index between two parameters of a formula were to be identified as significant, it could, for instance, signify that one of the parameters must be above or below a specific threshold in order for the other to be taken into account. This scenario would be incongruent with the objective of fetal weight estimation, as the larger the value of a biometric parameter is, the larger the EFW should be, irrespective of the value of the remaining parameters. Hence, since none of the formulas under investigation admitted a significant higher-order Sobol’ index, they are all well defined.

An explanation for this phenomenon may be the bounds of the parameters. As depicted in figure 3, the parameters have different magnitudes, which can lead to the dominance of some of them in interaction terms. An example of this is Woo (1986), in which EFW depends exclusively on the interactions of BPD, AC and FL, yet admits small higher order Sobol’ indices.

This study indicated that the uncertainty associated with the estimates is characterized by generally narrow 95% CIs, although exceptions were observed during the early and late stages of gestation.

One potential explanation for the broader estimates is the smaller number of data points available for analysis in those weeks. As can be seen in table 2, most of the datasets utilized do not provide data for the aforementioned stages of gestation. This results in the bootstrap estimate being less reliable, thereby increasing the uncertainty, due to the smaller sample size. Another potential reason for this could be the variability of the data in those weeks. During the early gestation, particularly between weeks 10 and 15, the fetus is still small, which can make accurate measurements of the parameters difficult [72], thus influencing the 10th and 90th percentile charts on which this study’s approach relies.

### 4.2. Interpretations of results

To facilitate the interpretation of the results, some examples are in order. The following examples use data from Papageorghiou *et al*. [62], and concern week 38, during which it is important to estimate fetal weight.

Assuming a fetus with the median biometric parameters, Hadlock III (1985) calculates the EFW to be 2990.54g*.* When HC lies at the 90th percentile, with the remaining parameters lying at the median, then the EFW is 3102.99g (3.75% increase). Doing the same for AC results in the EFW to be 3376.14g (12.88% increase), whereas for FL is 3123.26g (4.43% increase). The respective first-order Sobol’ indices for HC, AC and FL are 0.076, 0.82 and 0.105. It is obvious that the most substantial change occurs when AC is altered, followed by FL, and then HC.

Assuming a fetus with the median biometric parameters, Hadlock IV (1985) computes the EFW to be 3049.05g*.* When BPD lies at the 90th percentile, with the remaining parameters lying at the median, then the EFW is 3116.23g (2.22% increase). Doing the same for HC results in the EFW to be 3116.92g (2.22% increase), whereas for AC the result is 3444.67g (12.98% increase) and for FL is 3173.29g (4.07% increase). The respective first-order Sobol’ indices for BPD, HC, AC and FL are 0.027, 0.028, 0.854 and 0.09. It is evident that the most significant change occurs when AC is altered, followed by FL, and then BPD and HC, which both affect EFW similarly.

The results of the above practical examples demonstrate complete alignment with the findings of the present study.

### 4.3. Implications

The findings of the present study facilitate the development of personalized and adaptive techniques for the estimation of fetal weight. Different clinical situations can affect the accuracy of certain biometric measurements. For example, there are cases where the fetus’s head can be challenging to be measured, especially during the later stages of pregnancy [8–10]. Therefore, to minimize the error in predictions, it is recommended to use a formula with small first-order indices for the head parameters (BPD and HC), during the later stages of gestation. Additionally, the presence of insignificant parameters affects clinical situations, particularly in the ability to perform a rough but precise estimate in emergency contexts. For instance, a sonographer aiming to conduct an urgent assessment of the EFW utilizing INTERGROWTH-21 (2017) at around the 33rd week of gestation could derive predictions comparable to those obtained from measuring both abdominal AC and HC by relying solely on the AC measurement, while offering a rough estimate for HC or even using the median value from a pertinent percentile chart.

Furthermore, personalized medicine can be easier to be implemented, particularly for fetuses with anatomical anomalies or asymmetrical growth. For instance, in cases of skeletal dysplasia [73], clinicians should avoid formulas with a high first-order index for FL. Similarly, in hydrocephalus [74], reliance on formulas with a high first-order index for HC can lead to overestimations of the EFW. This level of adaptability guarantees that the EFW accurately represents the fetus’s actual condition, rather than being skewed by formulaic inaccuracies.

Another key implication of this study is the ability to refine training and quality control measures for sonographers. Assuming that a clinic uses some particular formulas for estimating fetal weight, and those formulas are highly dependent on a specific parameter, then training programmes should aim to improve sonographers’ skills in acquiring accurate and consistent measurements of that parameter.

### 4.4. Strengths and limitations

A notable strength of this study is its minimal reliance on a single, specific dataset of measurements, which could potentially be biased towards particular values, due to the ethnicity of the mother, operator variability and technical factors of ultrasound equipment, among others. On the contrary, it relies solely on the estimates of the 10th and 90th percentile charts, allowing it to investigate the entire parameter space evenly, ensuring that the results are objective and impartial. Furthermore, the results are based on a bootstrap estimate of numerous datasets, with participants of various ethnicities, which provides additional assurance regarding the reliability of the findings.

A limitation of this study is the exclusion of outlier measurements from the explored parameter range. The omission of measurements scoring below the 10th and above the 90th percentile ensures the validity of the results for appropriate for gestational age fetuses. However, it should be noted that the respective indices for small and large gestational age fetuses may vary from those presented in the current article. Hence, the dependence of fetal weight on individual biometric parameters for such cases could be explored in a future study. An additional limitation of this study is the Sobol’ method’s inability to account for parameter correlations. Despite the findings being in agreement with prior studies and being practically sound as outlined through §4, it would be valuable to explore the conclusions reached by utilizing alternative sensitivity analysis methods, such as Shapley effects [75], derivative-based global sensitivity measure [76], Borgonovo’s indices [77], etc., in a future study.

## 5. Conclusions

This study applied global sensitivity analysis using the Sobol’ method to evaluate how different biometric parameters influence the estimation of the fetal weight produced by utilizing a range of ultrasound formulas throughout pregnancy. Our findings demonstrate that not only does the importance of specific measurements change with gestational age, but it also varies significantly from one formula to another.

Across many formulas, AC generally stands out as a dominant parameter, especially during later gestation. However, the degree of reliance on AC—just like the rest of the parameters—differs among formulas. For instance, some formulas may be highly sensitive to changes in AC, while others might show a greater dependence on FL or even display a balanced contribution from several parameters during early gestation.

These variations imply that clinicians and sonographers should interpret fetal weight estimates in a stage-specific and formula-specific context. In practice, this means that when a particular formula is used, the corresponding sensitivity to its biometric inputs should guide both the measurement focus and the interpretation of the estimated fetal weight. Tailoring the approach in this way can improve the accuracy of assessments and enhance decision-making in prenatal care.

Future research could further investigate these differences and work towards refining existing formulas or developing new ones that utilize the methodology of this study to generate models, customized to address the unique characteristics of specific scenarios.

## Data Availability

Data and relevant code for this research work are stored in GitHub [78] and have been archived within the Zenodo repository [79]. Supplementary material is available online [80].

## References

[1] Carranza Lira S, Haro González LM, Biruete Correa B. 2007 Comparison between clinical and ultrasonographic measurements to estimate fetal weight during labor: a new clinical calculation formula. Ginecol. Y Obstet. De Mex **75**, 582–587. 18800576

[2] Chauhan S. 1998 Limitations of clinical and sonographic estimates of birth weight: experience with 1034 parturients. Obstet. Gynecol. **91**, 72–77. (10.1016/s0029-7844(97)00590-5) 9464724

[3] Hoopmann M, Abele H, Wagner N, Wallwiener D, Kagan KO. 2010 Performance of 36 different weight estimation formulae in fetuses with macrosomia. Fetal Diagn. Ther. **27**, 204–213. (10.1159/000299475) 20523027

[4] Mehdizadeh A, Alaghehbandan R, Horsan H. 2000 Comparison of clinical versus ultrasound estimation of fetal weight. Am. J. Perinatol. **17**, 233–236. (10.1055/s-2000-10003) 11110339

[5] Esinler D, Bircan O, Esin S, Sahin EG, Kandemir O, Yalvac S. 2015 Finding the best formula to predict the fetal weight: comparison of 18 formulas. Gynecol. Obstet. Investig. **80**, 78–84. (10.1159/000365814) 26183256

[6] Saltelli A, Tarantola S, Campolongo F, Ratto M. 2002 Sensitivity analysis in practice: a guide to assessing scientific models, 1st edn. New York, NY: Wiley.

[7] Saltelli A, Ratto M, Andres T, Campolongo F, Cariboni J, Gatelli D, Saisana M, Tarantola S. 2007 Global sensitivity analysis. the primer, 1st edn. New York, NY: Wiley.

[8] Molina FS, Terra R, Carrillo MP, Puertas A, Nicolaides KH. 2010 What is the most reliable ultrasound parameter for assessment of fetal head descent? Ultrasound Obstet. Gynecol. **36**, 493–499. (10.1002/uog.7709) 20533441

[9] Zeng Y, Tsui PH, Wu W, Zhou Z, Wu S. 2021 Fetal ultrasound image segmentation for automatic head circumference biometry using deeply supervised attention-gated V-Net. J. Digit. Imaging **34**, 134–148. (10.1007/s10278-020-00410-5) 33483862 PMC7887128

[10] Poojari VG, Jose A, Pai MV. 2022 Sonographic estimation of the fetal head circumference: accuracy and factors affecting the error. J. Obstet. Gynecol. India **72**, 134–138. (10.1007/s13224-021-01574-y) PMC9343492 35928073

[11] Dakwar Shaheen J, Hershkovitz R, Mastrolia SA, Charach R, Eshel R, Tirosh D, Shaheen N, Baron J. 2019 Estimation of fetal weight using Hadlock’s formulas: is head circumference an essential parameter? Eur. J. Obstet. Gynecol. Reprod. Biol. **243**, 87–92. (10.1016/j.ejogrb.2019.09.024) 31678760

[12] Sobol' IM. 2001 Global sensitivity indices for nonlinear mathematical models and their Monte Carlo estimates. Math. Comput. Simul. **55**, 1. (10.1016/s0378-4754(00)00270-6)

[13] Hammami A, Mazer Zumaeta A, Syngelaki A, Akolekar R, Nicolaides KH. 2018 Ultrasonographic estimation of fetal weight: development of new model and assessment of performance of previous models. Ultrasound Obstet. Gynecol. **52**, 35–43. (10.1002/uog.19066) 29611251

[14] Plonka M, Bociaga M, Radon-Pokracka M, Nowak M, Huras H. 2020 Comparison of eleven commonly used formulae for sonographic estimation of fetal weight in prediction of actual birth weight. Ginekol. Pol. **91**, 17–23. (10.5603/gp.2020.0005) 32039463

[15] Burd I, Srinivas S, Paré E, Dharan V, Wang E. 2009 Is sonographic assessment of fetal weight influenced by formula selection? J. Ultrasound Med. **28**, 1019–1024. (10.7863/jum.2009.28.8.1019) 19643784

[16] Geerts L, Widmer T. 2011 Which is the most accurate formula to estimate fetal weight in women with severe preterm preeclampsia? J. Matern. Fetal Neonatal Med. **24**, 271–279. (10.3109/14767058.2010.485232) 21231823

[17] Pinette MG, Pan Y, Pinette SG, Blackstone J, Garrett J, Cartin A. 1999 Estimation of fetal weight: mean value from multiple formulas. J. Ultrasound Med. **18**, 813–817. (10.7863/jum.1999.18.12.813) 10591444

[18] Dudley NJ. 2005 A systematic review of the ultrasound estimation of fetal weight. Ultrasound Obstet. Gynecol. **25**, 80–89. (10.1002/uog.1751) 15505877

[19] Siemer J, Egger N, Hart N, Meurer B, Müller A, Dathe O, Goecke T, Schild R. 2008 Fetal weight estimation by ultrasound: comparison of 11 different formulae and examiners with differing skill levels. Ultraschall Med. Eur. J. Ultrasound **29**, 159–164. (10.1055/s-2007-963165) 17602369

[20] Tonismae T, Voirol J, Schubert F, Shanks A. 2018 Comparison of formulas for estimation of fetal weight in pregnancies complicated by gastroschisis [34P]. Obstet. Gynecol. **131**, 182S–182S. (10.1097/01.aog.0000533208.66616.88)

[21] Combs CA, Jaekle RK, Rosenn B, Pope M, Miodovnik M, Siddiqi TA. 1993 Sonographic estimation of fetal weight based on a model of fetal volume. Obstet. Gynecol. **82**, 365–370. 8355935

[22] Ferrero A, Maggi E, Giancotti A, Torcia F, Pachì A. 1994 Regression formula for estimation of fetal weight with use of abdominal circumference and femur length: a prospective study. J. Ultrasound Med. **13**, 823–833. (10.7863/jum.1994.13.11.823) 7837327

[23] Hadlock FP, Harrist R, Sharman RS, Deter RL, Park SK. 1985 Estimation of fetal weight with the use of head, body, and femur measurements—a prospective study. Am. J. Obstet. Gynecol. **151**, 333–337. 3881966 10.1016/0002-9378(85)90298-4

[24] Halaska MG, Vlk R, Feldmar P, Hrehorcak M, Krcmar M, Mlcochova H, Mala I, Rob L. 2006 Predicting term birth weight using ultrasound and maternal characteristics. Eur. J. Obstet. Gynecol. Reprod. Biol. **128**, 231–235. (10.1016/j.ejogrb.2006.01.020) 16530919

[25] Hsieh FJ, Chang FM, Huang HC, Lu CC, Ko TM, Chen HY. 1987 Computer-assisted analysis for prediction of fetal weight by ultrasound-comparison of biparietal diameter (BPD), abdominal circumference (AC) and femur length (FL). Taiwan Yi Xue Hui Za Zhi J. Formos. Med. Assoc. **86**, 957–964. 3320270

[26] Stirnemann J *et al*. 2017 International estimated fetal weight standards of the INTERGROWTH-21^st^ Project. Ultrasound Obstet. Gynecol. **49**, 478–486. (10.1002/uog.17347) 27804212 PMC5516164

[27] Jordaan HVF. 1983 Estimation of fetal weight by ultrasound. J. Clin. Ultrasound **11**, 59–66. (10.1002/jcu.1870110202) 6404938

[28] Merz E, Lieser H, Schicketanz KH, Härle J. 1988 Intrauterine fetal weight assessment using ultrasound. a comparison of several weight assessment methods and development of a new formula for the determination of fetal weight. Ultraschall Med. (Stuttgart, Germany 1980) **9**, 15–24. (10.1055/s-2007-1011588) 3283926

[29] Ott W, Doyle S, Flamm S. 1986 Accurate ultrasonic estimation of fetal weight. Am. J. Perinatol. **3**, 193–197. (10.1055/s-2007-999866) 3718640

[30] Roberts AB, Lee AJ, James AG. 1985 Ultrasonic estimation of fetal weight: a new predictive model incorporating femur length for the low-birth-weight fetus. J. Clin. Ultrasound **13**, 555–559. (10.1002/1097-0096(199010)13:83.0.co;2-z) 3934218

[31] Schild RL, Fell K, Fimmers R, Gembruch U, Hansmann M. 2004 A new formula for calculating weight in the fetus of ≤ 1600 g. Ultrasound Obstet. Gynecol. **24**, 775–780. (10.1002/uog.1741) 15476297

[32] Shepard MJ, Richards VA, Berkowitz RL, Warsof SL, Hobbins JC. 1982 An evaluation of two equations for predicting fetal weight by ultrasound. Am. J. Obstet. Gynecol. **142**, 47–54. (10.1016/S0002-9378(16)32283-9) 7055171

[33] Thurnau GR, Tamura RK, Sabbagha R, Depp OR III, Dyer A, Larkin R, Lee T, Laughlin C. 1983 A simple estimated fetal weight equation based on real-time ultrasound measurements of fetuses less than thirty-four weeks’ gestation. Am. J. Obstet. Gynecol. **145**, 557–561. (10.1016/0002-9378(83)91195-X) 6829630

[34] Vintzileos AM, Campbell WA, Rodis JF, Bors-Koefoed R, Nochimson DJ. 1987 Fetal weight estimation formulas with head, abdominal, femur, and thigh circumference measurements. Am. J. Obstet. Gynecol. **157**, 410–414. (10.1016/S0002-9378(87)80182-5) 3618691

[35] Warsof SL, Gohari P, Berkowitz RL, Hobbins JC. 1977 The estimation of fetal weight by computer-assisted analysis. Am. J. Obstet. Gynecol. **128**, 881–892. (10.1016/0002-9378(77)90058-8) 888868

[36] Weiner C, Sabbagha R, Vaisrub N, Socol M. 1985 Ultrasonic fetal weight prediction: role of head circumference and femur length. Obstet. Gynecol. **65**, 812–817. 3889747

[37] Woo JS, Wan MC. 1986 An evaluation of fetal weight prediction using a simple equation containing the fetal femur length. J. Ultrasound Med. **5**, 453–457. (10.7863/jum.1986.5.8.453) 3528524

[38] Woo JS, Wan CW, Cho KM. 1985 Computer-assisted evaluation of ultrasonic fetal weight prediction using multiple regression equations with and without the fetal femur length. J. Ultrasound Med. **4**, 65–67. (10.7863/jum.1985.4.2.65) 3882988

[39] Dixit VK, Rackauckas C. 2022 GlobalSensitivity.jl: performant and parallel global sensitivity analysis with Julia. J. Open Source Softw. **7**, 4561. (10.21105/joss.04561)

[40] Jansen MJW. 1999 Analysis of variance designs for model output. Comput. Phys. Commun. **117**, 35–43. (10.1016/s0010-4655(98)00154-4)

[41] Araujo Júnior E, Martins Santana EF, Martins WP, Júnior JE, Ruano R, Pires CR, Filho SMZ. 2014 Reference charts of fetal biometric parameters in 31,476 Brazilian singleton pregnancies. J. Ultrasound Med. **33**, 1185–1191. (10.7863/ultra.33.7.1185) 24958405

[42] Brons JTJ, van Geijn HP, Bezemer PD, Nauta JPJ, Arts NFTh. 1990 The fetal skeleton; ultrasonographic evaluation of the normal growth. Eur. J. Obstet. Gynecol. Reprod. Biol. **34**, 21–36. (10.1016/0028-2243(90)90004-k) 2406167

[43] Browne P, Hamner L III, Clark W. 1992 Sonographic fetal growth curves from an indigent population in Atlanta, Georgia: I. Singleton pregnancies. Am. J. Perinatol. **9**, 467–476. (10.1055/s-2007-999291) 1418159

[44] Buck Louis GM *et al*. 2015 Racial/ethnic standards for fetal growth: the NICHD Fetal Growth Studies. Am. J. Obstet. Gynecol. **213**, 449.(10.1016/j.ajog.2015.08.032) PMC4584427 26410205

[45] Chitty LS, Altman DG, Henderson A, Campbell S. 1994 Charts of fetal size: 3. Abdominal measurements. BJOG **101**, 125–131. (10.1111/j.1471-0528.1994.tb13077.x) 8305386

[46] Chitty LS, Altman DG, Henderson A, Campbell S. 1994 Charts of fetal size: 2. Head measurements. BJOG **101**, 35–43. (10.1111/j.1471-0528.1994.tb13007.x) 8297866

[47] Chitty LS, Altman DG, Henderson A, Campbell S. 1994 Charts of fetal size: 4. Femur length. BJOG **101**, 132–135. (10.1111/j.1471-0528.1994.tb13078.x) 8305387

[48] Vega A de la, Ruiz-Febo N, Roberts ZC. 2008 Fetal ultrasound biometry: normative charts for a Puerto Rican population. P. R. Health Sci. J. **27**, 81–84. 18450238

[49] Dubiel M, Krajewski M, Pietryga M, Tretyn A, Breborowicz G, Lindquist P, Gudmundsson S. 2008 Fetal biometry between 20-42 weeks of gestation for Polish population. Ginekol. Pol. **79**, 746–753. 19140496

[50] Dulger O, Taser F, Osmanoglu UO, Serin AN. 2024 Fetal biometric parameter reference charts of a central anatolian Turkish population. Cureus **16**, e55252. (10.7759/cureus.55252) 38558579 PMC10981494

[51] Fouad M, Elsirgany S, Sharaf MF, Abdella RM, Sobh SM, Samir D, AbdelHakim NS. 2021 Clinical relevance of using local fetal biometry charts as compared to Intergrowth-21st standard: a cross-sectional study at Kasr El-Ainy Hospital, Cairo University, Egypt. Hong Kong J. Obstet. Gynaecol. **10**, 37515.

[52] Giorlandino M, Padula F, Cignini P, Mastrandrea M, Vigna R, Buscicchio G, Giorlandino C. 2009 Reference interval for fetal biometry in Italian population. J. Prenat. Med. **3**, 62–65. 22439050 PMC3279111

[53] Johnsen SL, Wilsgaard T, Rasmussen S, Sollien R, Kiserud T. 2006 Longitudinal reference charts for growth of the fetal head, abdomen and femur. Eur. J. Obstet. Gynecol. Reprod. Biol. **127**, 172–185. (10.1016/j.ejogrb.2005.10.004) 16289532

[54] Jung SI *et al*. 2007 Reference charts and equations of Korean fetal biometry. Prenat. Diagn. **27**, 545–551. (10.1002/pd.1729) 17431930

[55] Kiserud T *et al*. 2017 The World Health Organization fetal growth charts: a multinational longitudinal study of ultrasound biometric measurements and estimated fetal weight. PLoS Med. **14**, e1002220. (10.1371/journal.pmed.1002220) 28118360 PMC5261648

[56] Kwon JY, Park IY, Wie JH, Choe S, Kim CJ, Shin JC. 2014 Fetal biometry in the Korean population: reference charts and comparison with charts from other populations. Prenat. Diagn. **34**, 927–934. (10.1002/pd.4394) 24760468

[57] Lai FM, Yeo GS. 1995 Reference charts of foetal biometry in Asians. Singap. Med. J. **36**, 628–636. 8781636

[58] Lessoway VA, Schulzer M, Wittmann BK, Gagnon FA, Wilson RD. 1998 Ultrasound fetal biometry charts for a North American Caucasian population. J. Clin. Ultrasound **26**, 433–453. (10.1002/(sici)1097-0096(199811/12)26:9<433::aid-jcu3>3.0.co;2-o) 9800158

[59] Lindström L, Ageheim M, Axelsson O, Hussain-Alkhateeb L, Skalkidou A, Bergman E. 2020 Swedish intrauterine growth reference ranges of biometric measurements of fetal head, abdomen and femur. Sci. Rep. **10**, 22441. (10.1038/s41598-020-79797-8) 33384446 PMC7775468

[60] Munim S, Morris T, Baber N, Ansari Y, Iqbal Azam S. 2012 Growth charts of fetal biometry: a longitudinal study. J. Matern. Fetal Neonatal Med. **25**, 692–698. (10.3109/14767058.2011.592878) 21819339

[61] Paladini D, Rustico M, Viora E, Giani U, Bruzzese D, Campogrande M, Martinelli P. 2005 Fetal size charts for the Italian population. Normative curves of head, abdomen and long bones. Prenat. Diagn. **25**, 456–464. (10.1002/pd.1158) 15966062

[62] Papageorghiou AT *et al*. 2014 International standards for fetal growth based on serial ultrasound measurements: the Fetal Growth Longitudinal Study of the INTERGROWTH-21st Project. Lancet **384**, 869–879. (10.1016/s0140-6736(14)61490-2) 25209488

[63] Saksiriwuttho P, Ratanasiri T, Komwilaisak R. 2007 Fetal biometry charts for normal pregnant women in northeastern Thailand. J. Med. Assoc. Thai. **90**, 1963–1969. 18041409

[64] Stampalija T *et al*. 2020 Current use and performance of the different fetal growth charts in the Italian population. Eur. J. Obstet. Gynecol. Reprod. Biol. **252**, 323–329. (10.1016/j.ejogrb.2020.06.059) 32653605

[65] Verburg BO, Steegers EAP, De Ridder M, Snijders RJM, Smith E, Hofman A, Moll HA, Jaddoe VWV, Witteman JCM. 2008 New charts for ultrasound dating of pregnancy and assessment of fetal growth: longitudinal data from a population‐based cohort study. Ultrasound Obstet. Gynecol. **31**, 388–396. (10.1002/uog.5225) 18348183

[66] Casella G, Berger RW. 2024 Statistical inference. CRC Texts in Statistical Science Series. Boca Raton, FL: CRC Press.

[67] Campbell S, Wilkin D. 1975 Ultrasonic measurement of fetal abdomen circumference in the estimation of fetal weight. BJOG **82**, 689–697. (10.1111/j.1471-0528.1975.tb00708.x) 1101942

[68] Smith GCS, Smith MFS, McNay MB, Fleming JEE. 1997 The relation between fetal abdominal circumference and birthweight: findings in 3512 pregnancies. BJOG **104**, 186–190. (10.1111/j.1471-0528.1997.tb11042.x) 9070136

[69] Jazayeri A. 1999 Macrosomia prediction using ultrasound fetal abdominal circumference of 35 centimeters or more. Obstet. Gynecol. **93**, 523–526. (10.1016/s0029-7844(98)00520-1) 10214826

[70] Shi Cy, Zhang Xx, Jin Yz, Dong Y, Zhang Yy, Lin L, Li Xj, Zhang Br. 2005 Relationship between fetal abdominal circumference and birth weight: clinical findings in 1475 pregnancies. Zhonghua Fu Chan Ke Za Zhi **40**, 732–734. 16324244

[71] Turitz A, Quant H, Schwartz N, Elovitz M, Bastek J. 2013 Isolated abdominal circumference. Am. J. Perinatol. **31**, 469–476. (10.1055/s-0033-1353438) 23966127

[72] Salomon LJ *et al*. 2019 ISUOG practice guidelines: ultrasound assessment of fetal biometry and growth. Ultrasound Obstet. Gynecol. **53**, 715–723. (10.1002/uog.20272) 31169958

[73] Schramm T, Gloning KP, Minderer S, Daumer‐Haas C, Hörtnagel K, Nerlich A, Tutschek B. 2009 Prenatal sonographic diagnosis of skeletal dysplasias. Ultrasound Obstet. Gynecol. **34**, 160–170. (10.1002/uog.6359) 19548204

[74] Ambreen S, Ambreen U, Quratulain J. 2023 A study relating fetal hydrocephalus with family history and consanguinity in Pakistani population. Prof. Med. J. **30**, 157–162. (10.29309/TPMJ/2023.30.02.7286)

[75] Iooss B, Prieur C. 2017 Shapley effects for sensitivity analysis with correlated inputs:comparisons with Sobol’ indices, numerical estimation and applications. See https://arxiv.org/abs/1707.01334.

[76] Sobol’ IM, Kucherenko S. 2009 Derivative based global sensitivity measures and their link with global sensitivity indices. Math. Comput. Simul. **79**, 3009–3017. (10.1016/j.matcom.2009.01.023)

[77] Borgonovo E, Iooss B. 2017 Moment-independent and reliability-based importance measures. In Handbook of uncertainty quantification (eds R Ghanem, D Higdon, H Owhadi), pp. 1265–1287. Cham, Switzerland: Springer. (10.1007/978-3-319-12385-1_37)

[78] Tsilidis V. 2025 DopEFW.jl. GitHub. See https://github.com/TsilidisV/DopEFW.jl.

[79] Tsilidis V. 2025 TsilidisV/DopEFW.jl: initial release. Zenodo. (10.5281/zenodo.15210717)

[80] Bitsouni V, Gialelis N, Tsilidis V. 2025 Supplementary material from: Partial dependence of ultrasonically estimated fetal weight on biometric parameters. Figshare. (10.6084/m9.figshare.c.7837902) PMC12173515 40535947

